# Knockdown of NSD2 Suppresses Renal Cell Carcinoma Metastasis by Inhibiting Epithelial-Mesenchymal Transition

**DOI:** 10.7150/ijms.36128

**Published:** 2019-09-20

**Authors:** Xu Han, Lianhua Piao, Xiaofeng Yuan, Luhui Wang, Zhiwei Liu, Xiaozhou He

**Affiliations:** 1 Department of Urology, The Third Affiliated Hospital of Soochow University, 213003, Changzhou, China; 2 Institute of Bioinformatics and Medical Engineering, Jiangsu University of Technology, 213001, Changzhou, China; 3 Department of Orthopaedics, The Third Affiliated Hospital of Soochow University, 213003, Changzhou, China

**Keywords:** NSD2, renal cell carcinoma, metastasis, epithelial-mesenchymal transition

## Abstract

**Background:** Renal cell carcinoma (RCC) accounts for around 85% of all primary kidney neoplasms, which is one of top 10 common cancers worldwide. Nuclear receptor suppressor of variegation, enhancer of zeste, and trithorax (SET) domain-containing 2 (NSD2), belonging to NSD protein family, functions as an oncogene in the pathogenesis of multiple cancers.

**Methods:** GEO database was used to analyze the expression of NSD2 mRNA in renal cancer. Furthermore, NSD2 protein level in clear cell RCC (ccRCC) tissues was detected by immunohistochemistry (IHC). Knockdown efficiency of different siRNAs was evaluated by quantitative real-time PCR (qRT-PCR) and western blot analysis. The biological role and molecular mechanism of NSD2 in RCC metastasis were investigated via a series of functional experiments.

**Results:** NSD2 mRNA was massively amplified in several types of renal cancer, especially in metastatic ccRCC. The expression level of NSD2 protein was elevated in ccRCC tissues, but not correlated with pathological grading. The migratory and invasive properties were significantly repressed in NSD2-silenced RCC cells, concurrent with an increase of E-cadherin expression and a decrease of N-cadherin and Vimentin expression.

**Conclusion:** Down-regulation of NSD2 could potently suppress cell migration and invasion through inhibiting epithelial-mesenchymal transition (EMT), indicating that NSD2 may be a potential therapeutic target for metastatic RCC.

## Introduction

Kidney and renal pelvis cancer has become a serious health problem in the United States, with 73,820 new cases and 14,770 deaths estimated for 2019 1. Moreover, it ranks the 6^th^ and 8^th^ most prevalent cancer in male and female, respectively. Meanwhile, it was about 668,000 new cases and 234,000 deaths in China for 2015 2. Development of total or partial nephrectomy combined with immunotherapy can contribute to better overall survival for patients with localized neoplasms 3. Unfortunately, there is no effective therapeutic tool for patients with metastatic cancers at present. Since metastatic renal cell carcinoma (mRCC) is resistive to conventional radiotherapy and chemotherapy, it is imperative to discover novel therapeutic targets capable of improving outcomes of mRCC patients.

NSD2 (also known as WHSC1 and MMSET) was initially found to be deleted in Wolf-Hirschhorn syndrome (WHS) and rearranged with the immunoglobulin locus in 15%~20% multiple myeloma (MM) cases 4-5. Indeed, NSD2 gene, encoding two main isoforms, NSD2-long (1,365 amino acid) and NSD2-short (647 amino acid), was located at chromosome 4p16.3, which exhibits strong cancer relevance 6. Over the past decade, increasing evidence has revealed that NSD2 may be involved in epithelial-mesenchymal transition (EMT) in cancer metastasis. Over-expression of NSD2 could promote EMT process and invasive properties of prostate cancer 7. Contrarily, suppression of NSD2 by miRNAs would obviously attenuate cell growth and motility via impairing EMT process in gastric cancer and endometrial cancer 8-9. Nevertheless, the biological function and potential mechanism of NSD2 in metastatic progression of RCC remains undetermined.

In the current study, we found that the expression of NSD2 mRNA was extremely up-regulated in diverse types of renal cancer, particularly in metastatic ccRCC. Notably, NSD2 protein level was distinctly higher in ccRCC tissues than in normal tissues, but not associated with histological grading. In addition, depletion of NSD2 would greatly inhibit cell migration and invasion through repressing EMT process in RCC. Altogether, NSD2 may play an important role in RCC metastasis and targeting NSD2 may be a promising therapeutic approach for mRCC patients.

## Materials and methods

### GEO database reanalysis

The original NSD2 mRNA expression data was downloaded from GEO database. (Jones Renal, https://www.ncbi.nlm.nih.gov/geo/query/acc.cgi?acc=GSE15641) NSD2 mRNA expression between normal samples and different types of renal cancer samples was reanalyzed. Besides, NSD2 mRNA expression in primary ccRCC samples, metastatic ccRCC samples and paired adjacent normal samples was reanalyzed. (Jung Renal, http://www.ncbi.nlm.nih.gov/geo/query/acc.cgi?acc=GSE66270 and http://www.ncbi.nlm.nih.gov/geo/query/acc.cgi?acc=GSE66271)

### Immunohistochemistry (IHC) and ccRCC tissue microarrays

The expression of NSD2 protein in 65 ccRCC and 10 normal tissue sections were examined by IHC. Samples were obtained from patients with local or locoregionally advanced disease, who had never received any previous treatment. Slides of paraffin-embedded tissues were deparaffinized, rehydrated, and sections were treated at 96℃ for 20 minutes in antigen retrieval buffer. Anti-NSD2 (Abcam, dilution: 1:200) antibody was applied on tissue sections for 1 h incubation at room temperature. Then, the immune complexes were detected by using a DAB kit (MXB Biotechnologies, China). Semi-quantitative analysis of NSD2 staining using a 3-grade scale was defined as follows: negative, grade 0; mild, grade +1; and strong staining intensity, grade +2. Application of tissues was approved by the Ethics Committee of The Third Affiliated Hospital of Soochow University.

### Cell lines and cell culture

The RCC cell lines (786-O and ACHN) were purchased from Shanghai Cell Bank, Chinese Academy of Sciences. Cells were maintained in RPMI 1640 Medium (Gibco, USA) supplemented with 10% fetal bovine (BI, Israel), 100 U/ml penicillin and 100μg/ml streptomycin (Gibco, USA). Cells were incubated in a humidified atmosphere of 95% air and 5% CO_2_ at 37℃.

### Cell transfection

Three small interfering RNAs (siRNAs) targeting NSD2 were synthesized by GenePharma Company (Shanghai, China). RCC cells reaching to 50% confluence were transfected using Lipofectamine 2000 (Invitrogen, USA) according to manufacturer's protocols. The efficiency of transfection was evaluated by qRT-PCR after 48h and western blotting after 72 h, respectively.

### RNA isolation and quantitative real-time polymerase chain reaction (qRT-PCR)

Total RNA from cells was extracted with Ultrapure RNA Kit (DNase I) (CWBIO, China) according to manufacturer's instructions. The cDNA was synthesized using PrimeScript RT reagent Kit with gDNA Eraser (Takara, Japan), and qRT-PCR was conducted with TB Green Premix Ex Taq II (Takara, Japan) on ABI 7500 system (Applied Biosystems, USA). The GAPDH expression was used as a control and relative gene expression was calculated by the 2^-ΔΔCt^ method. The primers used for qRT-PCR were listed in Table 1.

### Transwell migration assay

Total 3×10^4^ cells in serum-free medium were plated in each insert (Millipore, USA). Complete medium with 10% FBS was added to the lower chambers. After incubating 24 h, cells remaining in the upper were removed. Cells on the bottom surface were fixed in 95% methyl alcohol and stained with 0.1% crystal violet (Beyotime, China). Migrating cells in five randomly selected fields were counted under an inverted microscope (Olympus, Japan).

### Transwell invasion assay

RCC cells (1×10^5^ cells per chamber) from each group were seeded with serum-free medium in the upper chambers pre-coated with 10% Matrigel (BD Biosciences, USA). The lower chambers were filled with complete medium containing 10% FBS. Cells remaining on the upper membranes were wiped away after incubation for 24 h. Invading cells on the bottom side of membranes were fixed and stained as the migration assay. Five random fields from each membrane were counted under an inverted microscope (Olympus, Japan).

### Western blot analysis

Cells were lysed with Whole Cell Lysis Assay kit (Keygen Biotech, China). Protein samples were electrophoresed in SDS-PAGE and then transferred to PVDF membranes (Millipore, USA). After blocked for 1 h with 5% skim milk at room temperature, the membranes were incubated with primary antibodies at 4℃ overnight. The membranes were washed thrice with TBST, and then incubated with secondary antibodies (Keygen Biotech, China). Signals were detected using an ECL system (Tanon, China) and quantified by ImageJ software. Mouse anti-NSD2 (Abcam, UK), rabbit anti-E-cadherin, anti-N-cadherin, anti-Vimentin (Cell Signaling Technology, USA) and rabbit anti-GAPDH (Keygen Biotech, China) were used in this study.

### Statistical analysis

All the experiments in this study were performed independently in triplicate and presented as mean ± standard deviation (SD). Statistical analyses were conducted by SPSS 20.0 software (IBM, USA). Differences between groups were calculated using Student's *t*-test, Mann-Whitney U test and Fisher exact test. P < 0.05 was considered to behave a statistically significant difference.

## Results

### NSD2 was up-regulated in renal cancer samples

**Figure 1:**
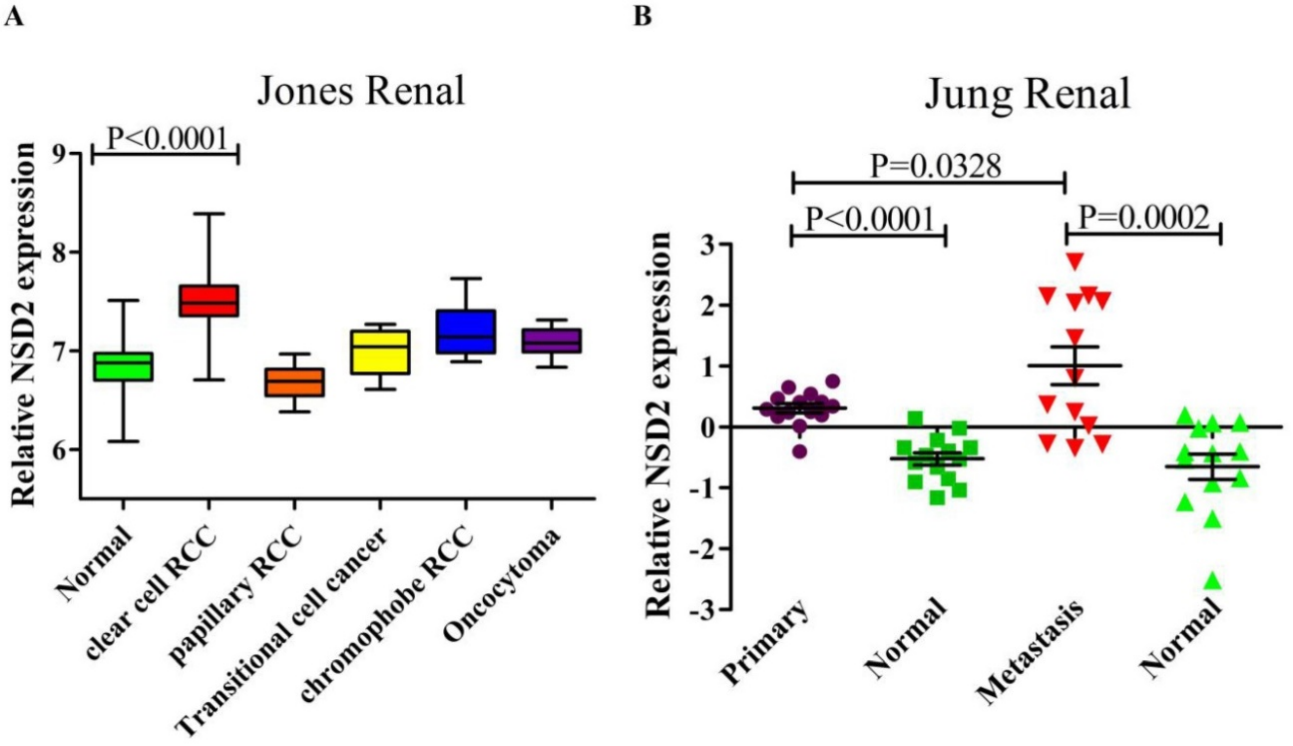
** NSD2 was up-regulated in renal cancer samples. (A)** NSD2 mRNA expression was elevated in several types of renal cancer and NSD2 was highly expressed in ccRCC (P < 0.0001). **(B)** The expression of NSD2 mRNA was obviously higher in primary ccRCC samples (P < 0.0001) and metastatic ccRCC samples (P = 0.0002). Besides, NSD2 mRNA in primary ccRCC expressed lower than in metastatic ccRCC (P = 0.0328).

The RNA sequencing data of renal cancer database (GSE15641) was reanalyzed, which contained 49 RCC samples, 20 non-RCC samples and 23 normal kidney samples. The result suggested that the expression of NSD2 mRNA was elevated in diversified types of renal cancer except papillary RCC (pRCC). Remarkably, NSD2 was highly expressed in ccRCC samples with the fold change = 1.544, comparing to normal samples (7.492±0.377 vs. 6.865±0.316, P < 0.0001, Figure 1A). Moreover, the RNA sequencing data from ccRCC databases (GSE66270 and GSE66271) was also reanalyzed. GSE66270 included 14 primary ccRCC tissues and 14 paired adjacent normal tissues, and GSE66271 included 13 metastatic ccRCC tissues and 13 paired adjacent normal tissues. The result showed that both in primary and metastatic ccRCC samples, NSD2 mRNA expression was obviously higher than in normal samples (P < 0.0001, P = 0.0002, Figure 1B). Comparing to metastatic ccRCC samples, NSD2 mRNA expressed lower in primary ccRCC samples (0.310±0.283 vs. 1.009±1.120, P = 0.0328, Figure 1B). Conclusively, these analyses implied that NSD2 might be implicated in RCC progression and metastasis.

### NSD2 was over-expressed in ccRCC tissues by immunohistochemistry (IHC)

**Figure 2:**
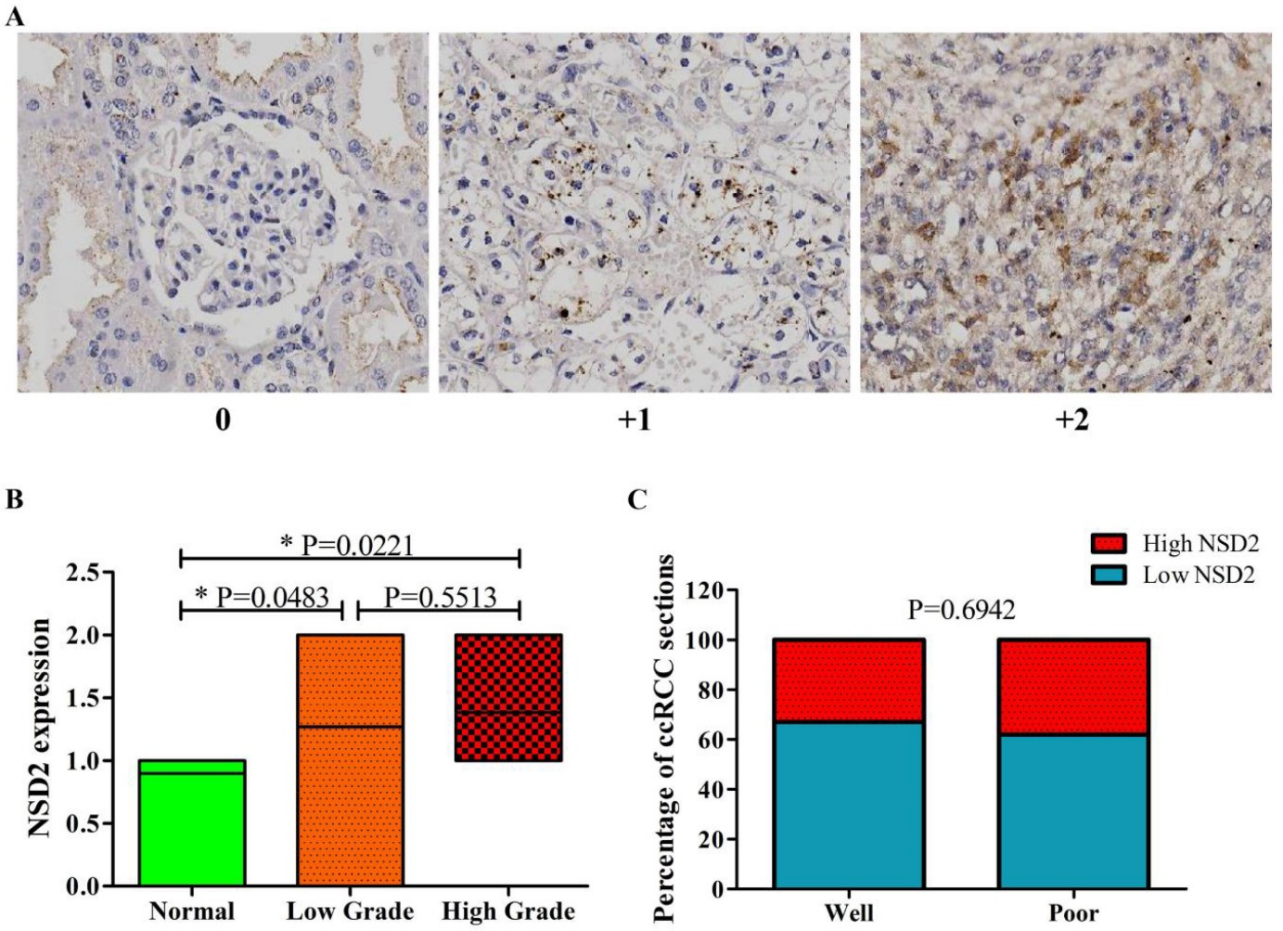
** NSD2 was over-expressed in ccRCC tissues by immunohistochemistry (IHC). (A)** IHC staining (3-grade score 0, +1, +2) for NSD2 in tissue microarrays of patients with locoregionally advanced ccRCC or normal epithelium. **(B)** NSD2 protein level was higher both in low grade and high grade ccRCC tissues than in normal tissues (P = 0.0483; P = 0.0221; Mann-Whitney U test). But the difference between low grade and high grade was not significant (P = 0.5513; Mann-Whitney U test). **(C)** High NSD2 protein level did not correlate with high grade in ccRCC tissues (P = 0.6942; Fisher exact test).

Next, the expression level of NSD2 protein was detected by IHC. Tissue microarrays including 65 spots from different patients with local or locoregionally advanced ccRCC and 10 normal epithelium tissues were used to examine NSD2 expression. Figure 2A showed representative results of IHC with 3-grade scale (IHC score 0, +1, +2). Intriguingly, the results of IHC showed that NSD2 protein level was significantly higher in ccRCC tissues (whatever low grade or high grade) than in normal tissues (P = 0.0483, P = 0.0221, Mann-Whitney U test, Figure 2B). However, there was no statistical significance between low grade and high grade (P = 0.5513, Mann-Whitney U test, Figure 2B).

The correlation of NSD2 expression with some clinical parameters, including gender, age and grade in 65 patients was investigated (Table 2). As results, gender and age were not associated with NSD2 expression. The percentage of ccRCC sections in low grade was similar to that in high grade (P = 0.6942, Fisher exact test, Figure 2C). Accordingly, our findings implied that NSD2 protein was over-expressed in ccRCC tissues, but not related to histological grading.

### NSD2 was effectively knocked down by siRNAs in RCC cells

**Figure 3:**
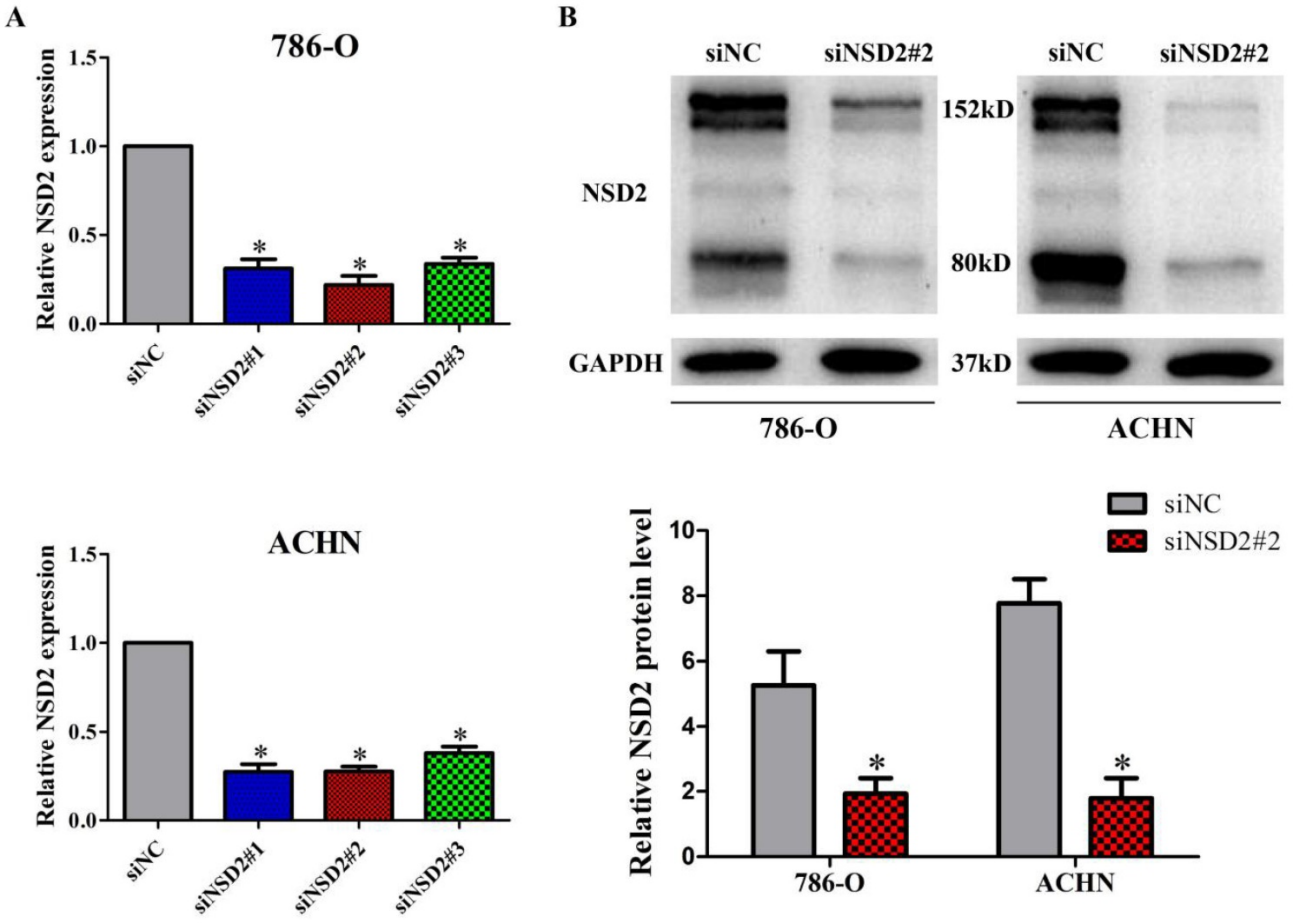
** NSD2 was effectively knocked down by siRNAs in RCC cells. (A)** The expression of NSD2 mRNA was potently suppressed by different siRNAs in 786-O and ACHN cells. **(B)** The expression of NSD2 protein was strikingly diminished by siNSD2#2 in 786-O and ACHN cells. (*, P < 0.05)

To investigate the biological role of NSD2 in RCC metastasis, we conducted the loss-of-function experiment. Three specific siRNAs (siNSD2#1, siNSD2#2 and siNSD2#3) were designed to reduce NSD2 expression. The results of qRT-PCR indicated that all the siRNAs could potently suppress NSD2 mRNA expression, and siNSD2#2 was the most effective one in 786-O and ACHN cells (all P < 0.05, Figure 3A). Additionally, the expression of NSD2 protein (NSD2-long, 152kD and NSD2-short, 80kD) was strikingly diminished by siNSD2#2 in transfected cells (both P < 0.05, Figure 3B). Hence, siNSD2#2 was chosen for further functional experiments.

### Depletion of NSD2 inhibited cell migration and invasion in RCC

**Figure 4:**
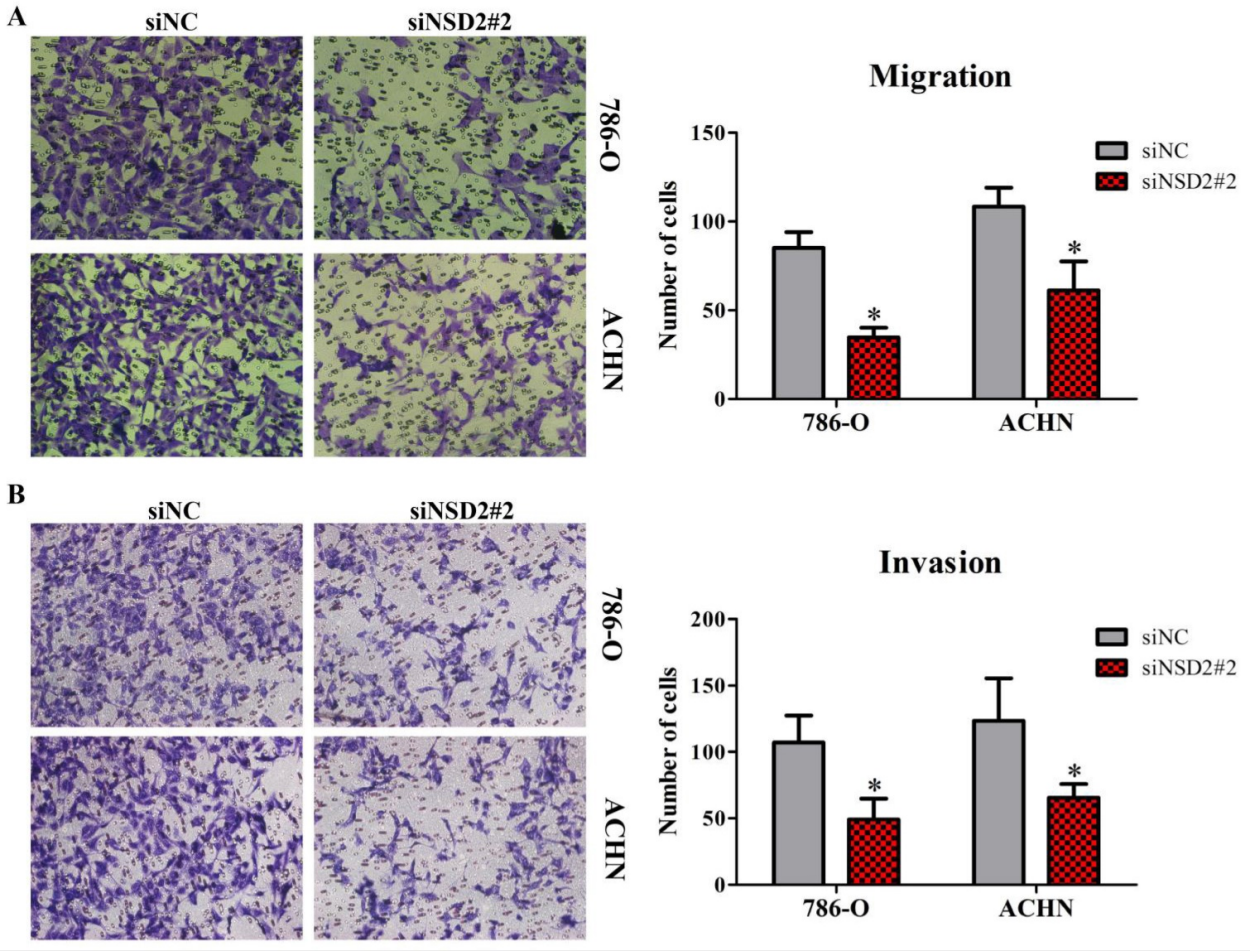
** Depletion of NSD2 inhibited cell migration and invasion in RCC. (A)** Down-regulation of NSD2 reduced migratory properties in 786-O and ACHN cells. **(B)** Sliencing of NSD2 repressed invasive properties in 786-O and ACHN cells. (*, P < 0.05)

Transwell migration and invasion assays were performed to identify the influence of NSD2 on metastatic potential of RCC cells. The results showed that down-regulation of NSD2 would lead to a conspicuous decrease of migratory abilities in 786-O and ACHN cells (both P < 0.05, Figure 4A). Besides, invasive abilities of RCC cells were also extremely repressed when NSD2 was silenced by siNSD2#2 (both P < 0.05, Figure 4B). Consequently, this study illustrated that knockdown of NSD2 could inhibit cell migration and invasion, indicating that NSD2 may be a vital regulator in the metastatic process in RCC.

### NSD2 regulated epithelial-mesenchymal transition (EMT)

**Figure 5:**
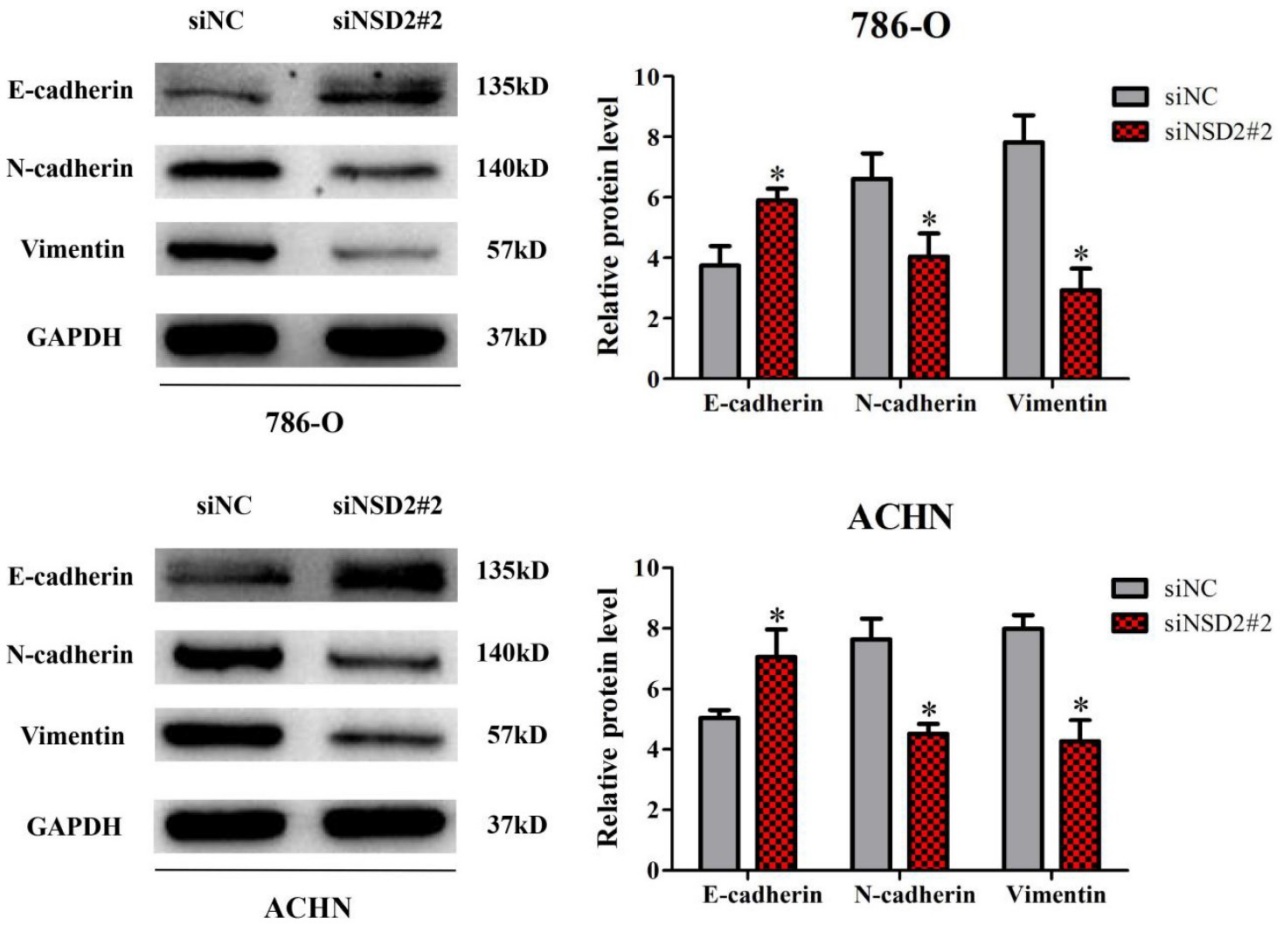
** NSD2 regulated epithelial-mesenchymal transition (EMT).** Inhibition of NSD2 increased the expression of E-cadherin protein and decreased the expressions of N-cadherin and Vimentin protein in 786-O and ACHN cells. (*, P < 0.05)

Previously, it has been reported that NSD2 would mediate cell migration and invasion via EMT process, which performed crucial functions in cancer progression and metastasis 7-9. Herein, western blot analysis was conducted to explore the molecular mechanism of NSD2 in RCC metastasis. After inhibition of NSD2, E-cadherin protein expression was increased in 786-O and ACHN cells, whereas the expressions of N-cadherin and Vimentin were decreased at protein level (all P < 0.05, Figure 5). The findings demonstrated that NSD2 could regulate EMT-related protein in RCC cells.

## Discussion

Renal cancer is an important cause of morbidity and mortality worldwide, with around 403,262 new cases and 175,098 deaths in 2018 10. Renal cell carcinoma (RCC) represents the most common subtype of all primary kidney neoplasms. Prior work has validated that histone lysine methyltransferases (HMTases) played critical roles in RCC progression and metastasis. Strikingly, metastatic RCC harbored higher rates of NSD1 inactivation and SETD2 mutation, compared with localized RCC 11. In addition, enhancer of zeste homolog 2 (EZH2) induced cell migration and invasion through suppressing E-cadherin expression in RCC 12. To our knowledge, there is no integrative investigation regarding to the biological function of NSD2 in RCC metastasis.

NSD2, as a pivotal chromatin remodeler, is a member of NSD protein family. The family members have been confirmed to involve in carcinogenesis through mono- or di-methylating lysine 36 of histone H3 (H3K36) 13-15. NSD2 preferentially catalyzed di-methylation of H3K36 (H3K36me2), which activates gene transcription and promotes tumorigenesis 16.

In prostate cancer, increased NSD2 led to the transformation of indolent prostate tumors to metastatic cancer 17. Besides, over-expression of NSD2 may induce cervical carcinogenesis by activating the AKT/MMP-2 signaling pathway 18. In the present study, we found that NSD2 mRNA was significantly amplified in several types of renal cancer by bioinformatic analysis, especially in metastatic ccRCC samples. Furthermore, NSD2 protein was over-expressed in ccRCC tissues, but not correlated with pathological grading, probably due to the limited number of cancer samples. Collectively, the results indicated that NSD2 served as an oncogene that distinctly facilitates RCC carcinogenesis.

Epithelial-mesenchymal transition (EMT), known to obtain migratory and invasive properties, is a vital process during cancer initiation and progression 19-20. Previous studies have manifested that aberrant expression of NSD2 would regulate EMT in diverse cancers 7-9. Meanwhile, EMT has been reported to be implicated in RCC metastasis 21-22. Nevertheless, the relationship of NSD2 expression and EMT process in metastatic RCC remains to be elucidated. Here, we discovered that knockdown of NSD2 would remarkably repress migration and invasion in RCC cells. Notably, E-cadherin protein expression was increased in NSD2-silenced cells, whereas N-cadherin and Vimentin expressions were decreased at protein level. Taken together, these findings demonstrated that silencing of NSD2 could potently suppress cell metastasis through inhibiting EMT in RCC. Nonetheless, more researches are necessary for further clarifying the detailed mechanisms between NSD2 and EMT, which may contribute to a better understanding of the involvement of NSD2 in metastatic RCC. Unfortunately, there is no effective inhibitor of NSD2 with high sensitivity and selectivity currently. Therefore, specific NSD2 inhibitors are urgently required to be discovered and investigated.

## Conclusion

In summary, this work firstly provided clinical and experimental evidence indicating the significance of NSD2 in RCC metastasis. Hence, targeting NSD2 may be a potential avenue in the treatment for patients with metastatic RCC.

## Figures and Tables

**Table 1:** All primers for qRT-PCR of NSD2 and GAPDH.

	Primer sequences
**NSD2**	
Forward	5'-AATATGACTCCTTGCTGGAGCAGG-3'
Reverse	5'-ATTTCAACAGGTGGTCTTTGTCTC-3'
**GAPDH**	
Forward	5'-AACGGATTTGGTCGTATTGGG-3'
Reverse	5'-CGCTCCTGGAAGATGGTGATG -3'
**NSD2 siRNAs**	
**① siNSD2#1**	
Sense	5'-CCCAGGAAAUGAAAGGGAUTT -3'
Antisense	5'-AUCCCUUUCAUUUCCUGGGTT -3'
**② siNSD2#2**	
Sense	5'-CCAGCUAAGAAAGAGUCUUTT -3'
Antisense	5'-AAGACUCUUUCUUAGCUGGTT-3'
**③ siNSD2#3**	
Sense	5'-GCCAGUAUCACGUACAGUUTT-3'
Antisense	5'-AACUGUACGUGAUACUGGCTT-3'
**Negative control (siNC)**	
Sense	5'-UUCUCCGAACGUGUCACGUTT-3'
Antisense	5'-ACGUGACACGUUCGGAGAATT-3'

**Table 2:** The relationship between NSD2 expression and clinicopathological parameters of ccRCC patients.

Clinicopathologic parameters	NSD2		P-value
	Low(0,+1)	High(+2)	
	(n=43)	(n=22)	
**Gender**			0.7590
Female	14	8	
Male	29	14	
**Age**			0.9224
<55	24	12	
≥55	19	10	
**Grade**			0.6942
Well differentiated	35	17	
Poorly differentiated	8	5	
